# Automatic MRI Volumetry Assisted Visual Assessment of the Medial Temporal Lobe in Clinical Dementia Work‐Up

**DOI:** 10.1002/brb3.70948

**Published:** 2025-09-30

**Authors:** Karin Persson, Hanneke F. M. Rhodius‐Meester, Trine Holt Edwin, Anne‐Brita Knapskog, Peter Bekkhus‐Wetterberg, Geir Selbæk, Knut Engedal, Till Schellhorn

**Affiliations:** ^1^ The Norwegian National Centre for Ageing and Health Vestfold Hospital Trust Tønsberg Norway; ^2^ Department of Geriatric Medicine Oslo University Hospital Oslo Norway; ^3^ Alzheimer Center Amsterdam, Neurology Vrije Universiteit Amsterdam, Amsterdam UMC Location VUmc Amsterdam the Netherlands; ^4^ Amsterdam Neuroscience, Neurodegeneration Amsterdam the Netherlands; ^5^ Faculty of Medicine University of Oslo Oslo Norway; ^6^ Division of Radiology and Nuclear Medicine Oslo University Hospital Oslo Norway

**Keywords:** Alzheimer's disease, cognitive impairment, dementia, NeuroQuant, visual rating scale

## Abstract

**Introduction:**

Efficient and cost‐effective diagnostic tools for supporting dementia assessment are increasingly important. We aimed to evaluate whether providing neuroradiologists with volumetric data from an automatic MRI software, NeuroQuant, enhanced the diagnostic accuracy of their visual MRI assessment.

**Methods:**

Two neuroradiologists assessed brain MRIs from 366 patients (mean age 67.5 years, SD 9.2, and 52% females) with subjective cognitive decline (SCD, *n* 79), mild cognitive impairment (MCI, *n* 86), or dementia (*n* 201). The MCI and dementia patients were further diagnosed according to an etiology of Alzheimer's disease (AD, *n* 217) versus non‐AD (*n* 70). In random order the neuroradiologists visually evaluated medial temporal lobe atrophy (MTA, scale 0–4) with and without having access to the NeuroQuant report of age and sex adjusted volumetric percentiles of the hippocampus. Receiver operating characteristics (ROCs) analyses were conducted to calculate the area under the curves (AUCs) for the visual MTA, the automated NeuroQuant percentile, and the combined NeuroQuant‐assisted MTA in discriminating dementia from SCD and AD from non‐AD.

**Results:**

The AUC of the visual MTA for dementia versus SCD discrimination increased slightly but not significantly when the neuroradiologists were provided with NeuroQuant results (AUC 0.76–0.79, *p* 0.28). Yet, the isolated NeuroQuant evaluation reached the highest accuracy (AUC 0.85, *p* < 0.001), significantly better than the MTA assessment (*p* 0.002) and the NeuroQuant‐assisted MTA (*p* 0.04). Only the isolated NeuroQuant assessment discriminated AD from non‐AD (AUC 0.60, *p* 0.006).

**Conclusion:**

On the basis of our findings, we suggest an increased use of clinically approved automatic volumetry methods in radiological departments.

## Graphical abstract

The aim was to evaluate whether providing neuroradiologists with volumetric data from an automatic MRI software, NeuroQuant, would enhance the diagnostic accuracy of their visual assessment. The AUC of the visual MTA for dementia versus subjective cognitive decline (SCD) discrimination increased, but not significantly, when NeuroQuant results were provided. Yet, the isolated NeuroQuant result reached the highest accuracy, significantly better than the isolated or the NeuroQuant‐assisted MTA. Only the isolated NeuroQuant assessment discriminated Alzheimer's disease etiology (AD) from non‐AD.

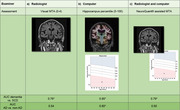

## 1 Introduction

The number of patients with dementia is expected to increase dramatically in the years to come, putting health care systems under pressure (World Health Organization 2023; Gjøra et al. 2021). In parallel, the demand for diagnostic procedures will increase. Additionally, future treatment options will require tools to enhance etiologic diagnosis and correct patient selection and close monitoring of possibly severe side effects (Cummings 2023; Filippi et al. 2023). Thus, the need for efficient and cost‐effective diagnostic methods and clinical supportive tools increases.

MRI of the brain has been regarded as a key instrument in the diagnostic work‐up of both neurodegenerative and vascular dementia etiologies (Frantellizzi et al. 2020; Frisoni et al. 2010). As one step of the standardized assessment, visual rating scales assessing brain regions known to be affected in various dementia etiologies have been thoroughly validated in both research and clinical settings (Wahlund et al. 2017; Ten Kate et al. 2017). Yet, the scales are still rather subjective, depending on the clinician's eye and experience, and require different cutoffs for different age groups (Harper et al. 2015; Rhodius‐Meester et al. 2017). Lack of training of radiologists and translation of research findings into clinical practice seem to have hindered widespread uptake in daily routine (Vernooij et al. 2019). To overcome these limitations, computer‐assisted methods, both various volumetric methods and AI‐based methods, have been developed. If automatic tools can aid the radiologist in the evaluation of regional changes, it could help alleviate the workload of the radiology services and enhance the accuracy of the radiological assessments, especially in situations where neuroradiological experience is limited. Automated MRI methods have been validated for research and are believed to be important tools to alleviate manual, effortful assessments. Yet these tools are still not widely incorporated into clinical workflow either, as stated by Vernooij et al. (2019). Further, automated methods have been shown to increase the degree of diagnostic confidence of physicians when added to the regular clinical assessments.

Several previous studies have found that providing neuroradiologists with computerized volumetric results improved the accuracy of detecting volume loss and increased diagnostic precision (Pemberton et al. 2021; Chagué et al. 2021; Vernooij et al. 2018). However, these studies were rather small, and not all used clinically accessible methods. Thus, more clinical validation is needed (Pemberton et al. 2021).

In the present study, we examined the effect of assisting a neuroradiologist with volumetric results from the clinically available automatic volumetry method NeuroQuant, which has previously been found to outperform visual assessments, in 2018 and 2024 (Persson et al. 2018, 2024). We hypothesized that the accuracy of the visual rating of medial temporal lobe atrophy (MTA) for separating dementia from subjective cognitive decline (SCD) and Alzheimer's disease (AD) from non‐AD would improve if the neuroradiologist was assisted by the NeuroQuant report of the same brain region.

## 2 Materials and Methods

### 2.1 Participants, Diagnoses, Patient Selection

Patients were recruited from the memory clinic at Oslo University Hospital (OUH) between 2010 and 2020. All patients had given written consent to be part of the Norwegian Registry of Persons Assessed for Cognitive Symptoms (NorCog), a national quality and research registry (Medbøen et al. 2022). As part of the clinical work‐up, patients are assessed with a structural MRI of the brain. In patients lacking a recent pre‐referral MRI examination (which is the case for approximately one‐third of the patients at the clinic), the MRI examination is usually performed at the hospital research scanner, and automatic brain volumetry using NeuroQuant is performed. Thus, 585 patients with available NeuroQuant results were eligible for inclusion in the present study. All patients were re‐diagnosed by two experienced physicians (K.P. and T.H.E.) based on all clinical data from the patient records, according to the following clinical criteria: the Jessen criteria for SCD and the NIA/AA (2011) criteria for mild cognitive impairment (MCI) and dementia (Jessen et al. 2014; McKhann et al. 2011; Albert et al. 2011). The underlying etiology of both MCI and dementia was diagnosed on the basis of the following clinical criteria: NIA/AA for AD (McKhann et al. 2011; Albert et al. 2011); McKeith criteria and Emre criteria for Lewy body and Parkinson's disease etiology (McKeith et al. 2017; Emre et al. 2007); Rascovsky criteria for frontotemporal dementia (FTD) (Rascovsky et al. 2011); and VASCOG (2014) criteria for vascular cognitive impairment (Sachdev et al. 2014). Findings from MRI regarding hippocampal volume or MTA are not included in any of the criteria for the various dementia diagnoses. The Mini‐Mental State Examination—Norwegian Revised Version 3 (MMSE‐NR3) was used as a descriptive measure of global cognitive function (score from 0 to 30, higher scores indicating better cognitive ability) (Strobel and Engedahl 2021).

**FIGURE 1:**
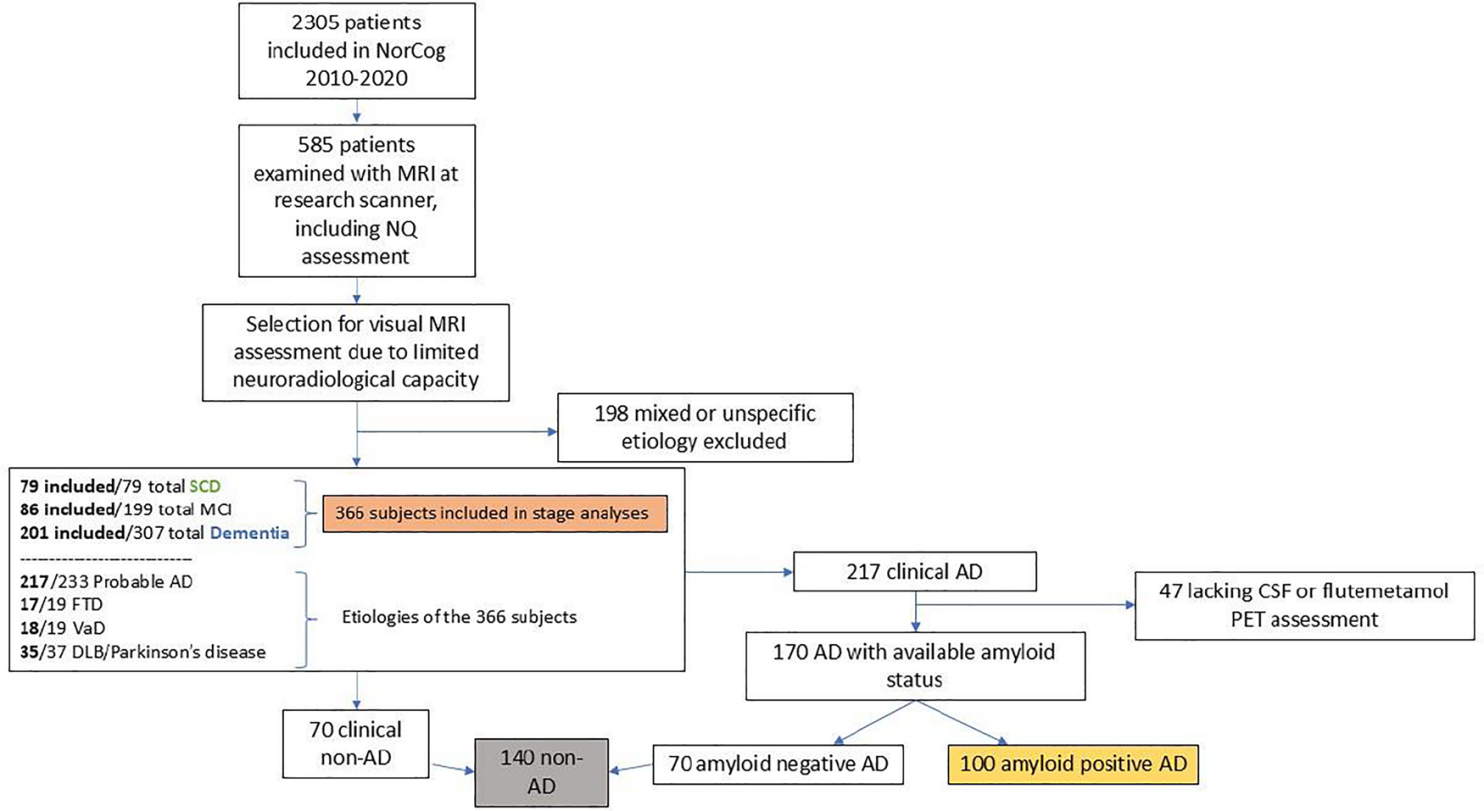
Flowchart illustrating patient selection for the stage analyses (orange) and stage categories SCD (green) and dementia (blue). Moreover, etiology of AD (yellow) and non‐AD (gray). AD, Alzheimer's disease; DLB, dementia with Lewy bodies; FTD, frontotemporal dementia; MCI, mild cognitive impairment; NorCog, the Norwegian Registry of Persons Assessed for Cognitive Symptoms; NQ, NeuroQuant; SCD, subjective cognitive decline; VaD, vascular dementia.

Due to a limited neuroradiologist capacity (financial limitations), not all 585 eligible patients could be included in the study. A flowchart of the patient selection is presented in Figure 1. First, all patients with a mixed or unspecific etiology were excluded. Second, all patients with SCD (*n* 79) were included, and among the remaining patients, a random selection of patients with a diagnosis of AD (68 with MCI and 149 with dementia) and non‐AD (18 with MCI and 52 with dementia) were included in the final study group (*n* 366). The MRI scans of these 366 subjects were assessed with visual ratings of MTA by two neuroradiologists. In comparison with the non‐included group, more patients in the included group were females (53% vs. 43%, *p* 0.042), and more of them had dementia (55% vs. 44%, *p* < 0.001). There was no significant age difference (*p* 0.403).

**All patients with a clinical diagnosis of AD that had been assessed with either a lumbar punction with assessment of CSF AD biomarkers or flutemetamol PET (referred to by clinical indication, *n* 170), that were amyloid positive, were regarded as amyloid‐verified AD according to Jack et al. (2024) (*n* 100). The 47 clinical AD patients lacking CSF or PET were excluded from the AD versus non‐AD analyses, as their biological AD status was unknown. On the contrary, amyloid‐negative clinical AD and all patients with another clinical diagnosis (regardless of their amyloid status) were grouped into the non‐AD group (*n* 140), Figure 1.

### 2.2 MRI Assessments

Three MRI assessments were to be compared: the visual MTA, the automated NeuroQuant percentile, and the combined NeuroQuant‐assisted MTA.

The MRI examinations were performed at the OUH core facility MRI research lab using a GE Signa HDxt 3T scanner (*n* 179) that was upgraded with new hardware in 2015 (GE Discovery MR750, *n* 169) and again in 2019 (GE Signa Premier, *n* 18). All system upgrades were carefully planned to maintain sequence parameter consistency to ensure fairly comparable imaging results across hardware upgrades.

The MRI scans of the 366 selected patients were assessed by two experienced neuroradiologists, blinded to any clinical information except for age and sex. The neuroradiologists were experienced in the use of visual rating scales to assess brain atrophy. Each patient received two visual assessments of MTA according to the Scheltens scale (Scheltens et al. 1992), one MTA assessment based on the images alone (*visual MTA*), and one MTA assessment in which the radiologist had been provided with the results of the automated NeuroQuant percentile of the hippocampus (*NeuroQuant‐assisted MTA*). The Scheltens scale is assessed on T1 sequences and evaluates atrophy of the medial temporal lobe on a scale from 0 to 4, where an increased score means more atrophy. Other MRI sequences were available as well but not used in the MTA evaluation. Volumetric analyses were produced by NeuroQuant (versions 1–3, CorTechs labs/University of California, San Diego, CA, USA). NeuroQuant produces a report, including both raw volumes of several brain regions and volumes adjusted to intracranial volume (ICV). Additionally, clinically useful percentiles in which the volumes have been compared to a norm set, including healthy individuals of different ages and sexs, are available (CorTechs Labs. Inc). For the present study, only the calculated hippocampus percentile was included (*NeuroQuant percentile*), and no data of other brain regions, as a proof‐of‐principle strategy.

**FIGURE 2:**
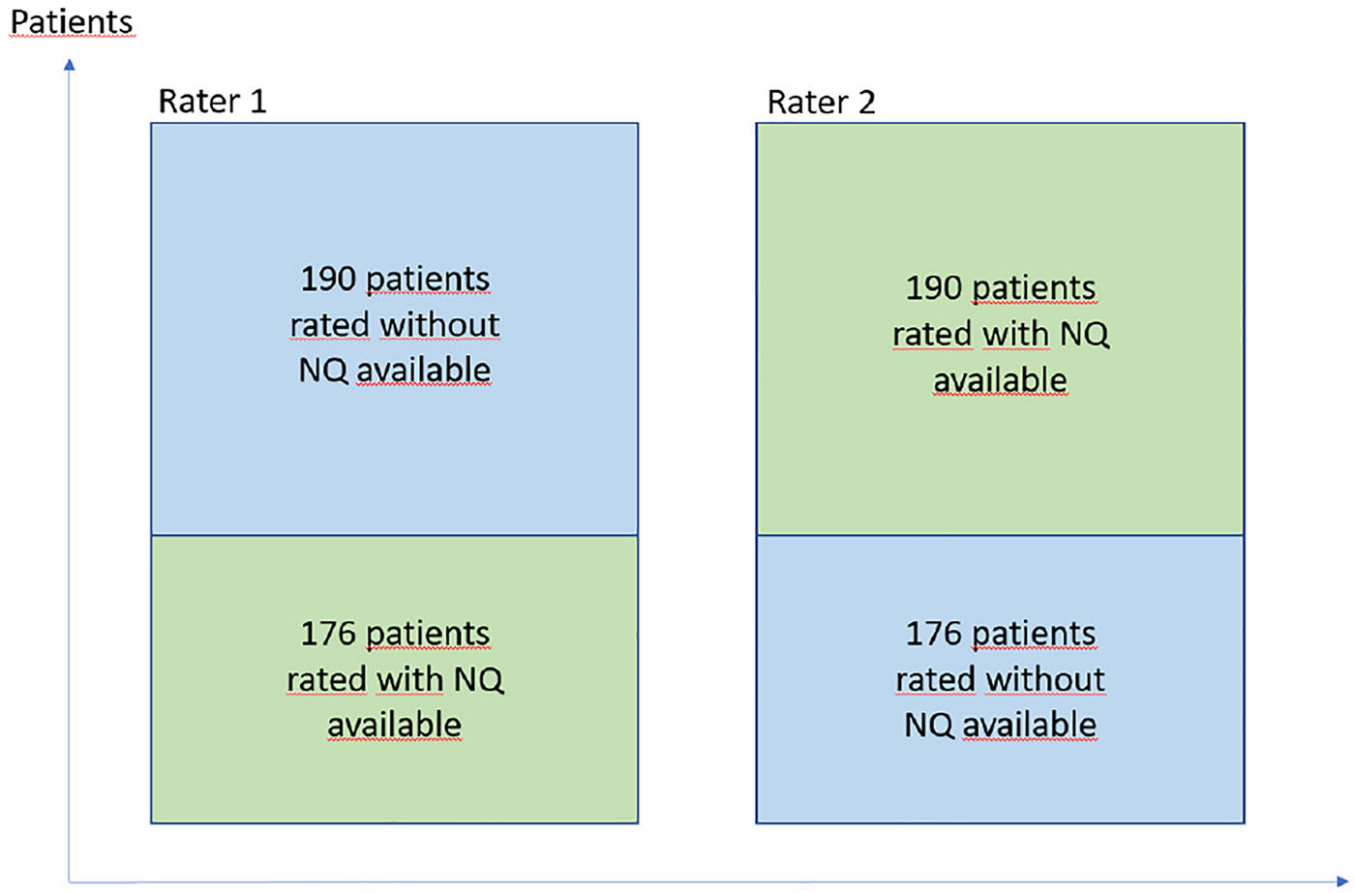
Visual rating procedure.

An inter‐rater reliability analysis of the visual MTA was performed in 42 patients, showing a weighted Cohen's kappa of 0.65. To avoid test–retest interference, each rater rated every patient only once. Thus, rater number one rated the first 190 patients without knowing the NeuroQuant result, and then he rated the remaining 176 patients having access to the NeuroQuant report. Rater number two rated the same patients but in the opposite direction, that is, first 190 patients having access to the NeuroQuant report, and the other 176 without knowing the NeuroQuant result. The rating procedure is visualized in Figure 2. Ratings were done for the left and right hemispheres separately, and the mean of the two sides was calculated and used in the analyses of diagnostic accuracy.

### 2.3 Amyloid Assessments

Analyses of cerebrospinal fluid AD core biomarkers (amyloid β42, phosphorylated^181^ tau, and total tau) had been performed at Akershus University Hospital, Norway, using ELISA technique with the Innotest kit (Innogenetics, Ghent, Belgium). Results were dichotomized as normal/abnormal based on cutoffs provided by the laboratory. For the present study, only the amyloid β42 status (and not tau statuses) was used as a marker of AD pathology as this enabled enhancement of the cohort by including patients that lacked CSF but that had an amyloid status from flutemetamol PET. Flutemetamol PET had been performed using the same PET/CT scanner, Siemens Biograph40 mCT (Siemens Healthineers, Erlangen, Germany), with a standard 18F‐Flutemetamol PET/CT protocol.

### 2.4 Statistics

Statistical analyses were performed using IBM SPSS Statistics for Windows (version 27, Armonk, NY, USA) and MedCalc Statistical Software version 22.026 (MedCalc Software Ltd., Ostend, Belgium; https://www.medcalc.org 2024) with a predefined significance level of 0.05. Student's *t*‐test, ANOVA, and *χ* ^2^ tests were used to describe differences between cognitive stage categories, that is, SCD, MCI, and dementia, and between etiologies, that is, AD and non‐AD. As age and educational level differed between cognitive stage categories, age‐ and education adjusted ANCOVA analyses were performed for the between‐group comparisons of descriptive MMSE results and MRI assessments. To validate the performance of the visual MTA, the automated NeuroQuant, and the NeuroQuant‐assisted MTA assessments in separating dementia from SCD and AD from non‐AD, receiver operating characteristics (ROCs) analyses with calculated area under the curve (AUC) were performed. To explore if the support of NeuroQuant was more beneficial in older versus younger patients, AUCs were also calculated for patients below/over age 65. The method of Hanley and McNeil (1983) was used to test if the differences between the AUCs of the dependent ROC curves (derived from the same cases) were of statistical significance (Hanley and McNeil 1983).

## 3 Results

Table 1 presents patient characteristics by cognitive stage categories. After adjusting for age and education, significant differences were found for all three MRI assessments between the cognitive stage categories. Table 2 presents characteristics by etiology. Here, only the automated NeuroQuant percentile was different between etiologies.

**TABLE 1:** Patient characteristics by cognitive stage categories.

	SCD (*n* 79)	MCI (*n* 86)	Dementia (*n* 201)	*p*
Age, years	62.0 (9.2)	67.3 (9.6)	69.7 (8.0)	**<0.001**
Females, *n*	44 (56%)	40 (47%)	108 (54%)	0.431
Education, years	15.0 (3.4)	14.1 (3.3)	13.0 (3.8)	**<0.001**
MMSE, score	29.2 (1.0)	27.8 (2.2)	23.0 (5.1)	<0.001*
Visual MTA (left), score	0.7 (0.8)	1.2 (0.9)	1.6 (1.0)	<0.001*
Visual MTA (right), score	0.8 (0.8)	1.1 (0.8)	1.6 (0.9)	<0.001*
NQ‐assisted MTA (left), score	0.6 (0.6)	1.0 (0.9)	1.5 (1.0)	<0.001*
NQ‐assisted MTA (right), score	0.7 (0.6)	1.1 (0.9)	1.6 (1.0)	<0.001*
NQ hippocampus percentile	62.1 (30.1)	39.9 (30.3)	20.1 (24.9)	<0.001*

*Note*: All continuous measures are expressed as mean (SD), and categorical measures as numbers (%). MTA scores are regarded as continuous. Between‐group comparisons with ANOVA, except *ANCOVA adjusted for age and education. Bold text indicating statistical significance.

Abbreviations: MCI, mild cognitive impairment; MMSE, mini mental status examination; MTA, medial temporal lobe atrophy; NQ, NeuroQuant; SCD, subjective cognitive decline.

**TABLE 2:** Patient characteristics by etiology.

	AD (*n* 100)	Non‐AD (*n* 140)	*p*
Age, years	68.7 (6.4)	68.7 (8.7)	0.494
Females, *n*	57 (57%)	69 (50%)	0.261
Education, years	13.1 (3.3)	13.3 (3.7)	0.365
MMSE, score	23.5 (5.7)	25.2 (4.1)	**0.008**
Dementia stage, *n*	81 (81%)	98 (71%)	0.054
Visual MTA (left), score	1.6 (1.0)	1.4 (1.0)	0.084
Visual MTA (right), score	1.5 (0.9)	1.4 (0.9)	0.218
NQ‐assisted MTA (left), score	1.4 (1.0)	1.4 (0.9)	0.460
NQ‐assisted MTA (right), score	1.4 (1.0)	1.4 (1.0)	0.491
NQ hippocampus percentile	20.1 (25.4)	29.2(28.5)	**0.005**

*Note*: All continuous measures are expressed as mean (SD), and categorical measures as numbers (%). MTA scores are regarded as continuous. Between‐group comparisons with Student's *t*‐test.

Bold text indicating statistical significance. Abbreviations: AD, Alzheimer's disease; MMSE, mini mental status examination; MTA, medial temporal lobe atrophy; NQ, NeuroQuant.

**FIGURE 3:**
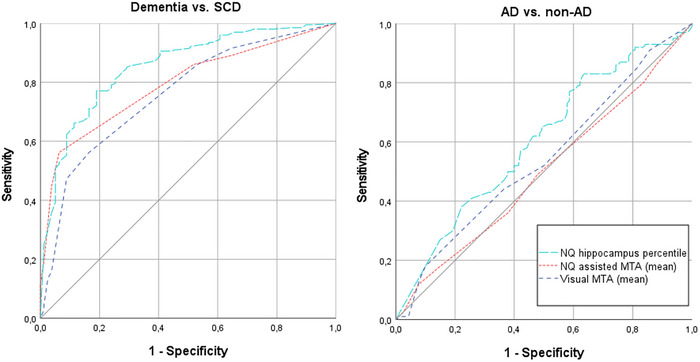
ROC curves of discriminating dementia from SCD (left) and AD from non‐AD (right). AD, Alzheimer's disease; MTA, medial temporal lobe atrophy; NQ, NeuroQuant; SCD, subjective cognitive decline.

To compare diagnostic validity of the three MRI assessments, ROC analyses were performed and are presented in Tables 3 and 4 and Figure 3. In dementia versus SCD discrimination (Table 3), the assessments, including NeuroQuant, and in particular the isolated NeuroQuant assessment, achieved the highest AUCs. Statistical comparisons of the AUCs of the three MRI assessments from Table 3 were performed. There was no difference between the AUCs of the visual MTA and the NeuroQuant‐assisted MTA (*p* 0.28). On the contrary, the isolated NeuroQuant assessment had a significantly higher AUC than both the visual MTA and the NeuroQuant‐assisted MTA (*p* 0.002 and *p* 0.04, respectively).

**TABLE 3:** Receiver operating characteristic (ROC) analyses, separating dementia from subjective cognitive decline (SCD).

	AUC	*p*	95% CI
Visual MTA (mean)	0.760	**<0.001**	0.699; 0.821
NQ‐assisted MTA (mean)	0.793	**<0.001**	0.740; 0.846
NQ hippocampus percentile	0.853	**<0.001**	0.805; 0.902

*Note*: MTA mean (mean of left and right side). Bold text indicating statistical significance.

Abbreviations: AUC, area under the curve; MTA, medial temporal lobe atrophy; NQ, NeuroQuant.

**TABLE 4:** Receiver operating characteristic (ROC) analyses, separating Alzheimer's disease (AD) from non‐AD.

	AUC	*p*	95% CI
Visual MTA (mean)	0.543	0.260	0.468; 0.617
NQ‐assisted MTA (mean)	0.498	0.956	0.423; 0.573
NQ hippocampus percentile	0.604	**0.006**	0.531; 0.676

*Note*: MTA mean (mean of left and right side). Bold text indicating statistical significance.

Abbreviations: AUC, area under the curve; MTA, medial temporal lobe atrophy; NQ, NeuroQuant.

Additionally, AUCs of discriminating SCD from MCI and MCI from dementia were calculated (Tables S1 and S2, Figures S1 and S2). Again, the isolated NeuroQuant assessment achieved the highest AUCs (0.70 and 0.71), but the difference against the other measurements was not of statistical significance (AUC 0.64 for visual MTA on both discriminations, and 0.65 and 0.66 for NeuroQuant‐assisted MTA).

In AD versus non‐AD discrimination (Table 4), only the isolated NeuroQuant assessment achieved a significant AUC; thus, no further comparative statistics between AUCs were performed.

Finally, AUCs separating dementia from SCD were calculated in subgroups of the patients below and above 65 years of age, Table 5. Incorporating NeuroQuant into the MTA evaluation only led to a significant increase of the AUC in the subgroup of patients above 65 years (*p* 0.022). Similar subanalyses in AD versus non‐AD distinction did not show any differences in the two age groups.

**TABLE 5:** Receiver operating characteristic (ROC) analyses of subgroups, separating dementia from subjective cognitive decline (SCD).

	AUC	*p*	95% CI
**Age ≤65 (51% dementia)**			
Visual MTA (mean)	0.722	**<0.001**	0.627; 0.817
NQ‐assisted MTA (mean)	0.689	**0.001**	0.589; 0.790
NQ hippocampus percentile	0.805	**<0.001**	0.721; 0.889
**Age >65 (85% dementia)**			
Visual MTA (mean)	0.678	**0.004**	0.567; 0.788
NQ‐assisted MTA (mean)	0.792	**<0.001**	0.711; 0.872
NQ hippocampus percentile	0.837	**<0.001**	0.769; 0.905

Bold text indicating statistical significance. Abbreviations: AUC, area under the curve; MMSE, mini mental status examination; MTA, medial temporal lobe atrophy; NQ, NeuroQuant.

**FIGURE 4:**
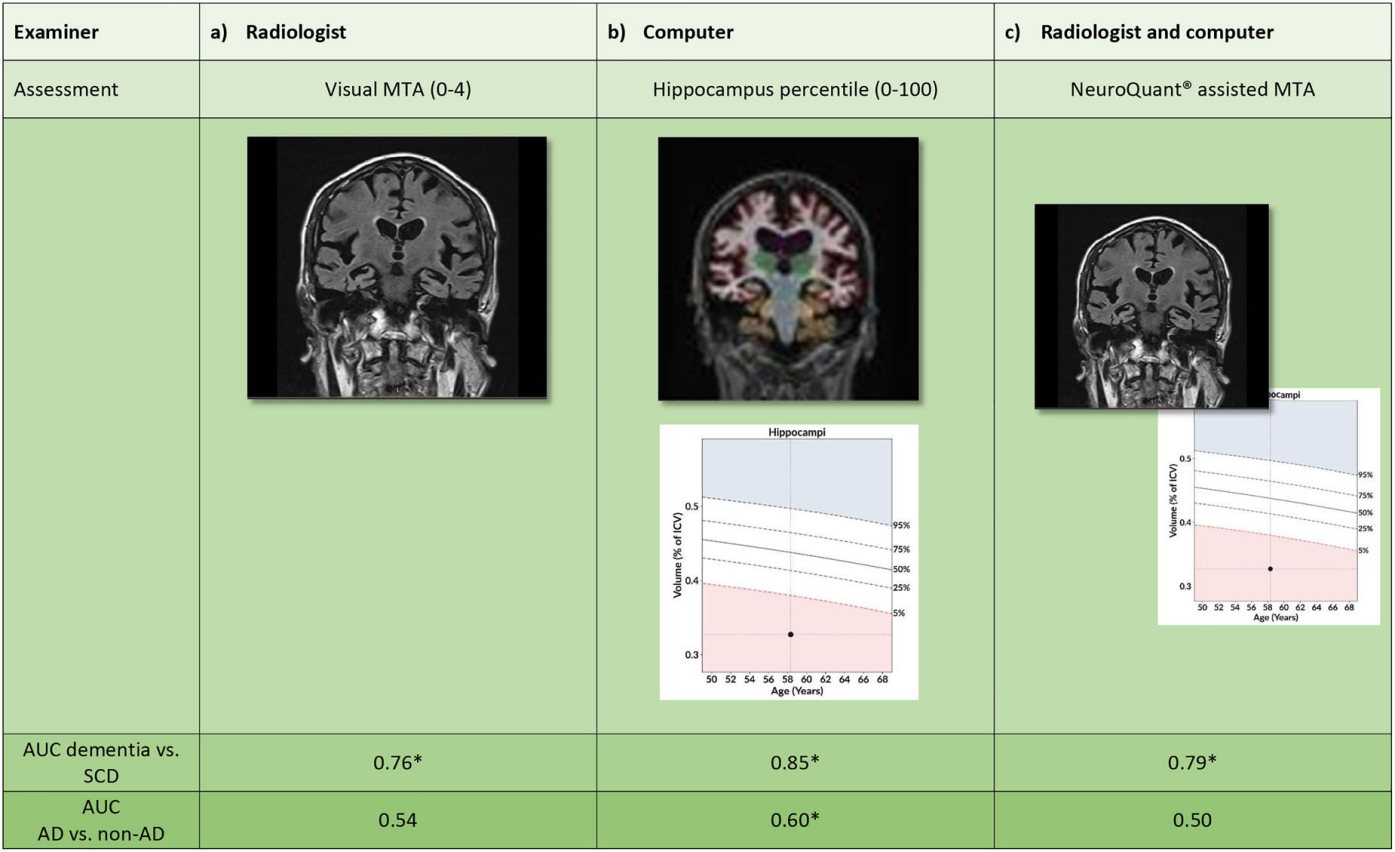
MRI assessments, *p* < 0.05 highlighted with an asterisk (*).AD, Alzheimer's disease; AUC, area under the curve; MTA, medial temporal lobe atrophy; SCD, subjective cognitive decline.

Figure 4 summarizes the main results of the study.

## 4 Discussion

A trend was observed indicating a slight, though statistically insignificant improvement in the ability to distinguish between dementia and SCD when neuroradiologists were assisted by the automated NeuroQuant report. The standalone NeuroQuant assessment achieved the highest accuracy in discriminating dementia from SCD and was the only assessment to reach statistical significance in distinguishing between AD and non‐AD. Subgroup analyses revealed that assisting neuroradiologists with NeuroQuant increased the AUC for separating dementia from SCD in patients aged 65 and older.

First, our hypothesis that assisting neuroradiologists with NeuroQuant volumetrics would improve diagnostic accuracy was not confirmed. Results from previous studies have supported the use of automated assessments to assist in visual evaluation in various situations and subgroups (Pemberton et al. 2021; Chagué et al. 2021; Vernooij et al. 2018). Vernooij et al. (2018) found automated quantification of several brain regions to improve the diagnostic accuracy for AD, but not for FTD. In a study by Chagué et al. (2021), machine learning support vector machine weight maps were shown to improve the diagnostic performance of junior but not senior radiologists, whereas a study by Pemberton et al. (2021) demonstrated that the combination of visual and volumetric assessments was primarily effective in experienced radiologists.** In our study, we did not compare the experience of the radiologists, because both neuroradiologists were equally senior and experienced. We believe the extensive clinical experience of the two neuroradiologists, and their relatively less extensive experience with this automatic method, made them less susceptible to influence by the new automatic method. Indeed, a European‐wide study by Vernooij et al. (2019) identified that only 6% of centers regularly used volumetric methods, and hurdles to the implementation of a new biomarker like this one included concerns about specificity and translation of research findings on group level to individual subjects. As the NeuroQuant percentile is an age and ICV corrected measure, one would expect an improved accuracy as visual assessments are known to be affected by age (Rhodius‐Meester et al. 2017). However, the visual MTA measure is, by design, not supposed to be affected by neither age nor the ICV, as it should only reflect the visual, and subjective, impression of the actual medial temporal lobe region. Thus, the rationale for radiologists to use such a percentile as a supporting tool is controversial. However, this was performed as a proof‐of‐principle to explore if the subjective MTA assessment would improve, as an indication of radiologists becoming more confident in their evaluation. Another possible explanation is that the MTA assessment includes more than just the volume of the hippocampus, assessing also the width of the temporal horn.

Second, our results showed that NeuroQuant alone achieved the highest AUC in both dementia versus SCD and AD versus non‐AD discriminations. Another study on the effect of adding automatic assessments to radiological reports also found the automatic classifier to be superior to the radiologist report (Chagué et al. 2021). This further aligns with findings from Koikkalainen et al. (2016), who reported a lower accuracy when MTA was incorporated into a comprehensive set of computerized quantification methods (Koikkalainen et al. 2016). However, our study focused only on the medial temporal lobe, whereas Kokkali et al. focused on several brain regions, which can explain the overall higher accuracy results of their automatic methods. The generally low accuracy results of the AD versus non‐AD discrimination in our study are not surprising considering that 30%–60% of patients, especially in the younger population with AD, do not exhibit hippocampal atrophy (Persson et al. 2022; Ferreira et al. 2020) and that hippocampal atrophy exists in other non‐AD etiologies (Hanseeuw et al. 2023). Moreover, including MCI patients further weakens the association between MTA and AD, as patients are at an earlier disease stage. Nevertheless, using isolated visual MRI to evaluate dementia etiology is inadequate and not according to current guidelines.

Finally, subgroup analyses revealed that the neuroradiologists appeared to benefit the most from having access to NeuroQuant in the age group above 65 years. This finding was somewhat unexpected, as younger patients typically present with milder atrophy and more often exhibit atypical atrophy patterns (Persson et al. 2022), which volumetric analyses should capture more effectively. Indeed, standalone NeuroQuant did demonstrate superior results in this younger age group. However, the results might imply that the radiologists were less prone to include the NeuroQuant result in their evaluations in the younger age group. On the other hand, as atrophy becomes more prevalent/common with age, distinguishing between MTA scores, such as 2 versus 3 or 3 versus 4, may have led the neuroradiologists to more frequently rely on the NeuroQuant percentile for assistance in this older age group. A power issue might also have affected the results, given a higher proportion of dementia in the older versus the younger age group.

There are limitations to this study. Only patients lacking a pre‐referral MRI were included in this study, as those are the patients being assessed with NeuroQuant. This could potentially introduce a selection bias. However, we believe the design of the study, with the aim being a comparison of assessments, should not be substantially affected by such a selection bias. The visual ratings were done by two radiologists; therefore, both inter‐ and intra‐rater reliability issues could have affected the result. The inter‐rater agreement was found substantial, and we believe the crossover rating procedure that was implemented should have diminished reliability issues as a bias of the results further. A strength of having two radiologists independently rate the patients was that it helped mitigate the risk of bias that could arise if a radiologist had previously seen the scans. It would have added to the study if the radiologists had different levels of experience, which was not the case. Finally, it should be emphasized that evaluation of MTA includes a larger region than only the hippocampus. As NeuroQuant gives the percentile of the hippocampus itself, our results could also indicate that isolated hippocampus atrophy is more strongly associated to AD and dementia than the somewhat larger region evaluated by the MTA.

To conclude, in general, the diagnostic accuracy of the visual evaluation of MTA was not significantly improved by providing the neuroradiologists with automated NeuroQuant percentiles, except in patients above 65. The isolated use of NeuroQuant did significantly better. Thus, a neuroradiological assessment of the medial temporal region does not add value if a quantitative assessment is available. We suggest automated volumetry as a valuable supplemental tool in clinical dementia diagnostic context, especially at centers lacking widespread experience using visual rating scales.

## Author Contributions

**Karin Persson** conceptualization, data curation, formal analysis, funding acquisition, investigation, methodology, project administration, resources, visualization, writing – original draft, writing – review & editing. **Hanneke F. M. Rhodius‐Meester** investigation, methodology, visualization, writing – review & editing. **Trine Holt Edwin** resources, writing – review & editing. **Anne‐Brita Knapskog** resources, writing – review & editing. **Peter Bekkhus‐Wetterberg** resources, visualization, writing – review & editing. **Geir Selbæk** data curation, investigation, project administration, writing – review & editing. **Knut Engedal** conceptualization, investigation, resources, writing – review & editing. **Till Schellhorn** conceptualization, data curation, formal analysis, investigation, methodology, resources, writing – review & editing

## Ethics Statement

All patients had consented to inclusion in the NorCog registry, including the use of data from examinations performed during the diagnostic work‐up, such as MRI, for research purposes. The study was approved by the Regional Committee of Medical Research Ethics of the South‐East Norway Regional Health Authority (REC South‐East number 2019/79).

## Conflicts of Interest

K.P. has contributed to clinical trials for Roche (BN29553) and Novo Nordisk (NN6535‐4730), outside the submitted work. A.B.K. has contributed to clinical trials for Roche (BN29553), Boehringer‐Ingelheim (1346.0023), Novo Nordisk (NN6535‐4730), and G.S.K. (219867). G.S. reports participation in Roche, Biogen, and Eisai advisory boards and has received honoraria for giving lectures at symposia sponsored by Eisai and Eli‐Lilly. H.R.M. has performed contract research for Combinostics, and all funding is paid to her institution. T.H.E. has contributed to clinical trials for Roche (BN29553), Boehringer‐Ingelheim (1346.0023), and G.S.K. (219867). The other authors declare that they have no conflicts of interest.

## Peer Review

The peer review history for this article is available at https://publons.com/publon/10.1002/brb3.70948.

## Supporting information




**Figure S1**: ROC curves of discriminating SCD from MCI.

**Figure S2**: ROC curves of discriminating MCI from dementia.

**Table S1**: ROC analyses, separating SCD from MCI

**Table S2**: ROC analyses, separating MCI from dementia

## Data Availability

The data that support the findings of this study are available on request from the corresponding author. The data are not publicly available due to privacy and ethical restrictions.
