# Supplementary figures and images for: Automatic MRI Volumetry Assisted Visual Assessment of the Medial Temporal Lobe in Clinical Dementia Work‐Up
Source: Brain Behav. 2025 Sep 30;15(10):e70948. doi: 10.1002/brb3.70948 (PMC12480925; doi:10.1002/brb3.70948)

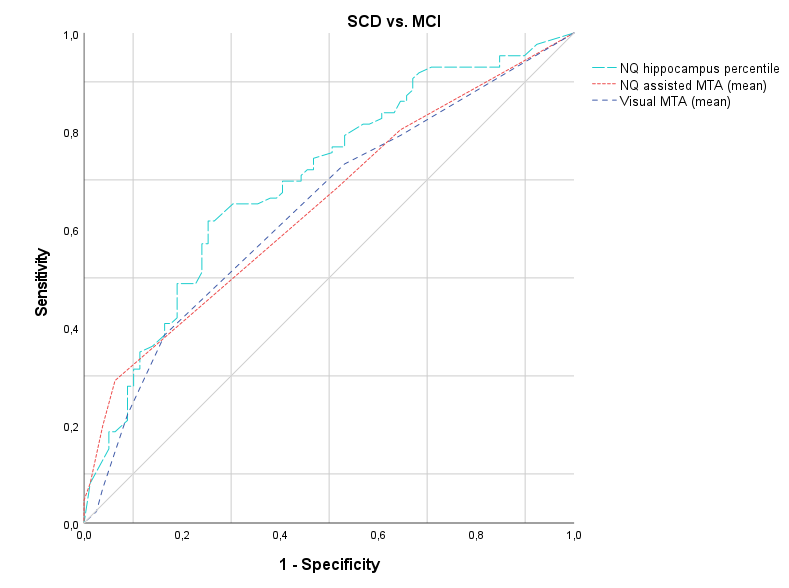


Suppl. figure 1. ROC curves of discriminating SCD from MCI.

Supplement: Supplementary file 1 — Figure S1: ROC curves of discriminating SCD from MCI. [file BRB3-15-e70948-s001.docx]

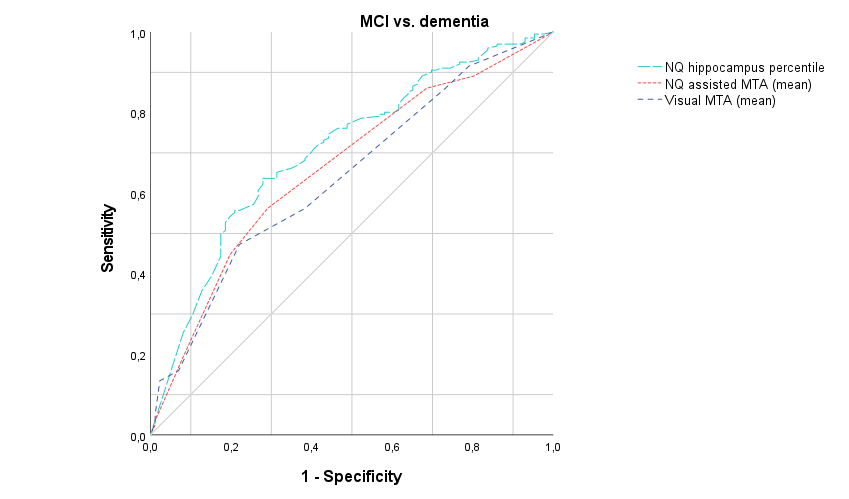


Suppl. figure 2. ROC curves of discriminating MCI from dementia.

Supplement: Supplementary file 2 — Figure S2: ROC curves of discriminating MCI from dementia. [file BRB3-15-e70948-s002.docx]
