# Supplementary material for: Automatic MRI Volumetry Assisted Visual Assessment of the Medial Temporal Lobe in Clinical Dementia Work‐Up
Source: Brain Behav. 2025 Sep 30;15(10):e70948. doi: 10.1002/brb3.70948 (PMC12480925; doi:10.1002/brb3.70948)
Supplement: Supplementary file 3 — Table S1: ROC analyses, separating SCD from MCI [file BRB3-15-e70948-s004.docx]

Suppl. table 1. ROC analyses, separating SCD from MCI

|  | AUC | p | 95% CI |
| --- | --- | --- | --- |
| Visual MTA (mean) | 0.640 | **0.002** | 0.556;0.725 |
| NQ assisted MTA (mean) | 0.647 | **0.001** | 0.563;0.730 |
| NQ hippocampus percentile | 0.699 | **<0.001** | 0.620;0.779 |
| *MTA: Medial temporal lobe atrophy; MTA mean (mean of left and right side); NQ: NeuroQuant®* | | | |
