# Supplementary material for: Automatic MRI Volumetry Assisted Visual Assessment of the Medial Temporal Lobe in Clinical Dementia Work‐Up
Source: Brain Behav. 2025 Sep 30;15(10):e70948. doi: 10.1002/brb3.70948 (PMC12480925; doi:10.1002/brb3.70948)
Supplement: Supplementary file 4 — Table S2: ROC analyses, separating MCI from dementia [file BRB3-15-e70948-s003.docx]

Suppl. table 2. ROC analyses, separating MCI from dementia

|  | AUC | p | 95% CI |
| --- | --- | --- | --- |
| Visual MTA (mean) | 0.640 | **<0.001** | 0.572;0.708 |
| NQ assisted MTA (mean) | 0.662 | **<0.001** | 0.595;0.730 |
| NQ hippocampus percentile | 0.706 | **<0.001** | 0.641;0.771 |
| *MTA: Medial temporal lobe atrophy; MTA mean (mean of left and right side); NQ: NeuroQuant®* | | | |
